# Changes in Starch In Vitro Digestibility and Properties of Cassava Flour Due to Pulsed Electric Field Processing

**DOI:** 10.3390/foods11223714

**Published:** 2022-11-18

**Authors:** Ladie Anne Conde, Biniam Kebede, Sze Ying Leong, Indrawati Oey

**Affiliations:** 1 Department of Food Science, University of Otago, Dunedin 9054, New Zealand; 2 Philippine Root Crop Research and Training Center (PhilRootcrops), Visayas State University, Baybay City 6521, Leyte, Philippines; 3 Riddet Institute, Palmerston North 4442, New Zealand

**Keywords:** pulsed electric fields, electric field strength, specific energy input, cassava flour, starch digestibility

## Abstract

The research aimed to investigate the effect of pulsed electric field (PEF) treatment on cassava flour at mild intensities (1, 2, and 4 kV/cm) combined with elevated levels of specific energy input (250–500 kJ/kg). Influences on starch digestibility, morphological characteristics, birefringence, short-range order and thermal properties were evaluated. Application of PEF at energy input no greater than 250 kJ/kg had negligible influence on the different starch digestion fractions of cassava flour but raised the rapidly digestible starch fraction at a combined electric field strength >1 kV/cm and energy input >350 kJ/kg. Morphological evaluation revealed that at this PEF combination, cassava starch’s external structure was consistently altered with swelling and disintegration, albeit some granules remained intact. Consequently, this led to disruption in the internal crystalline structure, supported by progressive loss of birefringence and significantly lower absorbance ratio at 1047/1022 cm^−1^. These physical and microstructural changes of the inherent starch promoted the shift in gelatinization temperatures to a higher temperature and reduced the gelatinization enthalpy. The study demonstrated that PEF can be utilized to change the starch fraction of cassava flour, which is driven by electric field strength and specific energy input, causing changes in the starch-related properties leading to increased digestibility.

## 1. Introduction

Pulsed electric field processing is an emerging technology that involves the application of high-intensity electric pulses for a short duration (nano to microseconds) inside a treatment chamber confined between electrodes [1,2]. A significant number of studies have demonstrated that the remarkable advantages of PEF treatment include the low processing temperature, continuous processing nature, short treatment time, and uniform treatment intensity [3,4]. Depending on the intensity applied, it has been shown to improve the pressing yield of juices and extractability of valuable bioactive compounds, as well as inactivate enzymes and pasteurize liquid foods [2,5]. Commercially, PEF was found to be more economical than high-pressure processing in orange juice production [6]. In addition, industrial scale application of PEF pre-treatment of potatoes for french fry production revealed lower energy consumption, beneficial processing efficiency [7], and increased the crust hardness of fries [8].

PEF also has the potential to modify the microstructural and functional properties of biomacromolecules, such as starches [9]. Physical modification of starches using PEF has garnered scientific interest since the process is simple and fast, without involving chemical reagents, produces no residues and thus can result in “clean-label” products [1,10]. Previous work revealed that PEF technology can alter the physicochemical and functional properties of starch when treated as a suspension or solid-state, with a previous study demonstrating that potato starch (B polymorph) had less structural resistance to increasing electric field intensity than wheat or pea starch (A and C polymorph, respectively) at solid-state [4,11,12,13]. These authors also found that at a high-intensity electric field strength (30–50 kV/cm) and energy input range of 28.85–80.14 kJ/kg [4], partial gelatinization of starch was induced despite the temperature of starchy suspension not reaching the gelatinization temperature (<50 °C) after PEF treatment. This was supported by the presence of gel-like structures, loss of birefringence, and reduced crystallinity and gelatinization enthalpy, along with morphological damages of starch.

There have been recent attempts to utilize PEF in starch-rich matrices and to understand their impact on inherent starch, e.g., potato [14], black beans [15], rice grains [16], and even on fine powders such as flour [17]. As starch, in its native form, has limited functionality, physical modification by PEF can change not only its properties but also the quality of the food material as a whole. For instance, PEF pre-treatment increased the hardness of cooked rice [18] and the outer crust of potato fries [8], while it increased the chewiness of thermally processed black beans [19]. However, the digestibility of their inherent starch had varied responses; starch hydrolysis was enhanced in rice [18], while the influence of PEF pre-treatment was minimal in black beans [15] and potato fries [8] compared to other processing factors considered in their study. In the case of ground material such as flour, the initial investigation of PEF use was for bacterial reduction in dark rye flour [20], but recent work revealed the opportunity to create novel starchy products with various properties. As an example, PEF-treated oat flour had increased pasting stability, producing a paste with reduced syneresis and hardness [17] and the probability of creating customized “oat fractions” with targeted physicochemical properties [21]. Naturally fermented sorghum flour after PEF treatment had increased porosity of the cellular membrane, thereby increasing the release of phenolics bound by the protein–carbohydrate matrix and enhanced the health properties of sorghum flour [22]. Besides oat and sorghum, no other research work has looked at the effect of PEF on inherent starch and flour quality from other botanical sources.

Cassava, for example, is a highly valued agricultural crop in the developing countries of Asia, South America, and Africa. Compared to other crops such as rice, corn, and sorghum, cassava produces more energy per hectare [23]. Its underground tuber can be made into flour that is predominantly composed of starch (67 to 88%), fiber (1–5%), and in lower quantities protein, fat, and ash [24,25]. Compared to its highly exploited starch (tapioca) fraction, utilization of whole cassava flour is mostly in baked products and a few in alcohol production [26,27]. Hence, PEF can be explored as a prospective non-thermal, chemical-free, and environmentally safe technology to modify the properties of cassava flour through its inherent starch and create information for potential new applications.

To have a better understanding of the effect of PEF on cassava flour, key processing parameters previously identified to have a dominant influence on starch properties, electric field strength (EFS) and specific energy input (SEI) [17,28], were considered. Therefore, the study aimed to investigate the effect of the combination of different levels of mild electric field strengths and elevated specific energy inputs on the in vitro digestibility, morphological, microstructural and thermal properties of PEF-treated cassava flours.

## 2. Materials and Methods

### 2.1. Materials

Flour from Philippine cassava cultivar NSIC Cv-44 was kindly provided by PhilRootcrops of the Visayas State University, Leyte, Philippines, and had a starch content of 85.83%. *Aspergillus oryzae* α-amylase (30 U/mg) and porcine pancreas pancreatin (P1750, 4 × USP) was manufactured by Sigma, St. Louis, MO, USA. Porcine stomach pepsin (A4289, 0.7 FIP-U/mg) was from AppliChem, Barcelona, Spain and porcine bile extract (SC-214601) from ChemCruz, Dallas, TX, USA. The GOPOD reagent and total starch kit was purchased from Megazyme, Wicklow, Ireland.

### 2.2. PEF System

PEF treatment was carried out using the ELCRACK^®^ HVP 5 PEF system (German Institute of Food Technologies, Quakenbrück, Germany) using a batch configuration. The chamber consisted of two parallel stainless-steel electrodes with a 40 mm gap. Square wave pulses were supplied at a constant pulse width of 20 μs with a 100 Hz pulse frequency. This was monitored using an oscilloscope (Model UT2025C, Uni-Trend Group Ltd., Dongguan City, China) during each treatment.

Three electric field strengths (1, 2, and 4 kV/cm) combined with four levels of specific energy inputs (250, 350, 450, and 500 kJ/kg) were selected for this study. These combinations induced notable progressive physical changes (turbidity and viscosity) with no or minimal electrical arcing based on preliminary trials. The pulse count and energy delivered by the system was used to calculate the total specific energy input according to Alpos et al. [19] using Equation (1):

(1) $$SEI\;\left(\mathrm{kJ}/\mathrm{kg}\right)=\frac{Pulse\;energy\;delivered\;\left(\mathrm{kJ}\right)\times Pulse\;number}{Total\;weight\;\left(\mathrm{kg};\;flour\;and\;water\right)}\;$$

### 2.3. PEF Treatment of Cassava Flour Suspension

Flour suspension was prepared by weighing 1.05 g of cassava flour into the PEF chamber and then 33.95 g of cold distilled water was added to make a 3% (*w*/*w*) flour suspension. The temperature of the suspension was kept between 8–15 °C to ensure actual pulse energy was maintained especially at higher specific energy input requirements. To guarantee a similar level of water imbibition, a delay of 3 min was enforced from water addition to flour, mixing and start of PEF treatment [17]. Three replicates were prepared for each processing condition, in which each replicate was from a pool of 10 independent PEF-treated samples. Untreated suspensions were also made for comparison, hereafter referred as “control”. The temperature (Table 1) and conductivity of the suspensions were recorded before and after treatment using a handheld conductivity meter (CyberScan CON 11, Eutech Instruments, Singapore). Initial conductivity recorded for cassava flour suspension ranged from 654–683 μS/cm.

### 2.4. In Vitro Digestibility

Digestibility of starch in cassava flour samples was evaluated using a 3-stage static in vitro digestion method according to Minekus et al. [29] with modifications. Flour samples (0.5 g) were placed in a 100 mL Schott bottle and hydrated with a recorded amount of ultrapure water 1 h prior to analysis.

#### 2.4.1. Preparation of Digestion Solutions

The solutions were prepared according to the work of Abduh et al. [14]. “Saliva juice” was prepared by mixing 0.117 g (22 mM) NaCl, 0.149 g (2 mM) KCL, and 2.1 g (25 mM) NaHCO_3_ in 1 L ultrapure water. A α-amylase solution was prepared by mixing 0.0125 g α-amylase per millilitre of ultrapure water. The gastric solution was prepared by adding 8.8184 g (151 mM) NaCl and 2.1 g KCL (28 mM) in 1 mM HCl (pH 3). To a 100 mL of gastric solution, 4 g porcine stomach pepsin was added to make the “gastric juice”. The “intestinal juice” was prepared by adding 1 g of porcine pancreas pancreatin and 0.8452 g of porcine bile extract into 100 mL of 0.1 M NaHCO_3_ (pH 7). All solutions with enzymes were prepared on the day of the assay and kept chilled until use. Additionally, 1 M HCl and 1 M NaOH were also prepared.

#### 2.4.2. In Vitro Digestion Procedure

For stage 1 or the oral phase, the hydrated flour samples were mixed with 8 mL saliva juice and incubated at 37 °C (Contherm Scientific Ltd., Hutt City, New Zealand) for 5 min on a shaker (DLAB, SK-R1807-S, Hong Kong) with a rocking motion (55 strokes/min). Afterwards, 2 mL of α-amylase solution was added and incubated for another 5 min. Then, the pH of the solution was adjusted to 3 with 1 M HCl to deactivate the amylase. In stage 2 or the gastric phase, the acidic mixture was added with 8 mL gastric juice and incubated for 120 min with rocking. Aliquots of 0.5 mL were collected at 0, 60, and 120 min, with immediate heat shocking in boiling water for 10 min for enzyme deactivation. Then, pH was adjusted to 7 with 1 M NaOH to deactivate the pepsin. For stage 3 or the intestinal phase, 16 mL of intestinal juice was added into the neutralized digest and incubated with rocking. Aliquots of 0.5 mL were again collected at 0, 20, 40, 60, 90, 120, 180, and 240 min, followed by immediate heat shocking. All the collected digests were added with 2.5 mL of 100 mM sodium acetate buffer (pH 5), vortexed, and stored at 4 °C until glucose analysis which was conducted within 24 h.

#### 2.4.3. Measurement of Hydrolyzed Starch

The diluted digests were centrifuged at 2056× *g* (Beckman GPR Centrifuge, Brea, CA, USA) for 20 min at 20 °C. Supernatant aliquots of 50 μL, which contain all the glucose released during enzymatic hydrolysis, were transferred into microtubes. To this, 1.5 mL of GOPOD reagent was added, mixed, and microtubes were incubated in a water bath (Grant, Cambridge, UK) at 50 °C for 20 min. The absorbance of the mixture was measured using a microplate reader at 510 nm (BioTek^®^ Synergy™ 2, Winooski, VT, USA) and the glucose produced was calculated against a 100 mM sodium acetate buffer (pH 5) blank and a glucose standard (1 mg/mL in 0.2% benzoic acid, Megazyme, Wicklow, Ireland). Starch digestibility was assessed through the determination of the percentage of rapidly digestible starch (RDS), slowly digestible starch (SDS) and resistant starch (RS) produced. These were calculated according to Equations (2)–(4):

(2) $$\mathrm{RDS}\left(\%\right)=\frac{\left(G_{20}-FG\right)\times 0.9}{TS}\times 100$$

(3) $$\mathrm{SDS}\left(\%\right)=\frac{\left(G_{120}-G_{20}\right)\times 0.9}{TS}\times 100$$

(4) $$\mathrm{RS}\left(\%\right)=\frac{TS-\left(\mathrm{RDS}+\mathrm{SDS}\right)}{TS}\times 100$$

where *G_20_* and *G_120_* is the amount of glucose released during intestinal starch hydrolysis at 20 min and 120 min, respectively; *FG* is the amount of inherent free glucose in the flour; 0.9 is an adjustment to convert glucose to anhydroglucose (as occurs in starch); and *TS* is the total starch content of the flour [30]. The initial free glucose and total starch content of flour samples were determined using the Megazyme K-TSTA kit.

#### 2.4.4. Kinetic Modelling of In Vitro Starch Digestibility at the Small Intestinal Phase

To estimate the rate of starch digestion through the amount of glucose released in the whole digest per gram (dry basis) of flour during the small intestinal phase, a linear regression model was used (Equation (5)) [31]:

(5) $$C=\left(k\times t\right)+C_{0}$$

where *C* is the amount glucose released at digestion time *t*, *C_0_* is the amount of glucose at the start of the small intestinal phase (0 min), and *k* is the rate constant of starch digestion through glucose release (min^−1^). The model fitting and estimation of kinetic parameter *k* was estimated using R software (v.4.0.4 2021) and R Studio (v.1.4.1103 2021, Boston, MA, USA). The goodness of fit of the kinetic models were evaluated by their R^2^ value and through residual (random distribution of error) and parity plots.

### 2.5. Polarized Light Microscopy

Flour suspensions were diluted to 0.05% (*w*/*v*) with ultrapure water, transferred to glass slides, and viewed under the Olympus BX41-P microscope (Olympus, Tokyo, Japan) at 400× magnification. The micrographs were taken by an attached Canon EOS 1100D camera, while polarized images were captured through a rotatable polarizer (U-AN360P, Olympus).

### 2.6. Fourier-Transform Infrared Spectroscopy—Attenuated Total Reflection (FTIR-ATR) Analysis

The FTIR spectra of cassava flour samples were obtained by using a Bruker Optics FTIR Spectrometer (Alpha System, Billerica, MA, USA) with an ATR platinum diamond one accessory. Cleaning of ATR crystal with isopropanol-soaked delicate wipes and background scanning were performed prior to each sample scan. The spectrum was scanned from 400 to 4000 cm^−1^ with an accumulation of 32 scans at 4 cm^−1^ resolution. Baseline of the generated spectra were corrected using the OPUS software (Version 8.1, Bruker Optik, Ettlingen, Germany). Then, the spectral region of 1200–875 cm^−1^, associated with the short-range ordered structure of starch, was deconvoluted based on the second-order derivative peak identification [21,32] using the OMNIC software (Thermo Scientific Fisher Inc., Waltham, MA, USA). The ratio of the peak height of the absorbances around 1047 and 1022 cm^−1^ was used to quantify the ordered and amorphous structure of the flour’s starch [33,34,35], while the absorbance ratio of 1022/995 was used to measure the molecular order of starch double helices inside the crystallites [33,36].

### 2.7. Differential Scanning Calorimetry (DSC)

In a hermetically sealed aluminum pan, approximately 5 mg of flour was dispersed with distilled water (1:3 *w*/*v*) and allowed to stand for at least 2 h at room temperature prior to analysis. Using the TA Instruments Q2000 differential scanning calorimeter (New Castle, DE, USA), samples were equilibrated at 25 °C for 1 min then heated to 130 °C at a 5 °C/min rate with an empty sealed pan as a reference and a nitrogen flow of 50 mL/min. The transitional thermal properties were determined using the TA Instruments Universal Analysis 2000 Version 4.5A software (New Castle, DE, USA); onset, peak, conclusion temperatures, and gelatinization enthalpy (ΔH). The temperature range of gelatinization (Trange) was calculated using Equation (6) [37], while the degree of gelatinization (DG) was calculated as the percentage of ΔH relative to the untreated flour.

(6) $$T_{range}\left({}^{\circ}C\right)=2\times \left(T_{peak}-T_{onset}\right)$$

### 2.8. Statistical Analysis

Results are presented as mean value from 3 replicates ± standard deviation. One-way ANOVA was performed to compare the means of the studied parameters for the untreated and treated flours at a 5% significance level using SPSS version 28 (IBM Corp., Armonk, NY, USA), while Tukey’s HSD was used for post hoc analysis. If assumptions were not met, appropriate non-parametric alternative tests were used, i.e., Welch’s ANOVA with Games–Howell post hoc tests.

## 3. Results and Discussion

### 3.1. Changes in PEF Processing Parameters and Physical Properties of Cassava Flour Suspension upon PEF Treatment

The actual electric field strength and total specific energy input supplied by the PEF system are shown in Table 1. A slight decrease in EFS and progressive temperature increase were recorded with increasing SEI. Temperature increases with the rise in pulse number, energy input, and electric field strength, as a consequence of Joule heating [16,38]. Simultaneously, the rise in sample temperature causes increased electrical conductivity. In turn, it lowers the electrical resistance of the treatment chamber and may lead to a decrease in applied field strength [38]. Hence, for ease in designating samples, electric field strengths ~1, ~2, and ~4 kV/cm will be referred to henceforth as PEF 1, 2, and 4, respectively.

The conductivity increase after PEF treatment is mainly due to membrane electroporation and ion diffusion into the medium [38], but the cassava flour samples showed no or a smaller conductivity increase (0–9.97 μS/cm) compared to thermally processed oat flour (41–62 μS/cm) [21] and glutinous rice grain (40–140 μS/cm) [16] when treated at 2–4 kV/cm with 50–451 kJ/kg of SEI and at 3 kV/cm for 100–300 pulses, respectively. Presumably, the comminuted nature of cassava flour (pre-sieved in the miller at *ϕ* 0.25 mm) allows almost complete dispersion of ions when prepared as a suspension in the water prior to PEF treatment.

### 3.2. Effect of PEF on In Vitro Digestibility of Cassava Starches in PEF Treated Cassava Flours

**Figure 1:**
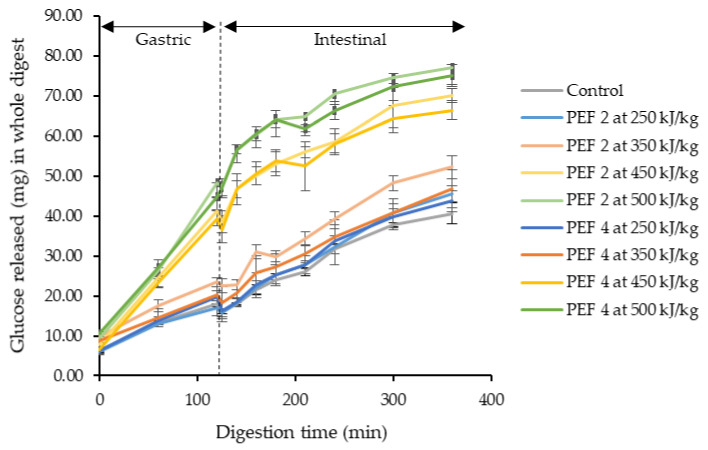
Glucose (mg) released in the whole digest per gram (dry basis) of “control” and selected PEF-treated cassava flours during the gastric and intestinal phases.

Figure 1 shows the extent of starch hydrolysis of untreated and PEF-treated cassava flours during the gastric phase (2 h) and small intestinal phase (4 h). One of the distinct observations was the increase of glucose released during the gastric phase. This suggests continued amylolytic activity during the gastric phase. An earlier study by Rosenblum et al. [39] revealed that salivary-type amylase is protected in a simulated gastric environment by starch and its end products, even retaining 24% of its activity in the presence of pepsin at pH 3 after 120 min. A substantially higher amount of glucose was released for both PEF 2 and 4 at 450–500 kJ/kg in the gastric phase and such increase continued during the 4 h long small intestinal phase. This revealed an increased susceptibility to digestive enzymes at higher EFS and SEI. During the small intestinal phase, the starch digestion rate constant (*k*) of the control and PEF-treated cassava flours was estimated (Table 2). Despite the higher amount of starch digested during the gastric phase for PEF 2 and 4 at 450–500 kJ/kg, upon entering the small intestinal phase where continued hydrolysis and absorption of nutrients normally occur, starch in PEF-treated cassava flour was digested at a similar rate to that of the control sample.

The extent and rate of starch digestibility of cassava flour in the human small intestine is also indicative of its glycemic response [40]. Englyst et al. [30] nutritionally classified them into rapidly digestible starch (RDS), slowly digestible starch (SDS) and resistant starch (RS) [30]. RDS is likely to be digested quickly in the small intestine, SDS is digested at a slower rate, while RS is not digested in the small intestine and is available for fermentation in the colon. In conjunction with the enhanced starch hydrolysis that occurred during the gastric phase for cassava flour treated at PEF 2 and 4 with 450–500 kJ/kg, these flour samples were found exhibiting a higher proportion of RDS and reduced RS fraction during small intestinal digestion (Table 2). An increase in the RDS fraction was not observed in other cassava flour samples PEF-treated at an energy input below 450 kJ/kg. Clearly, the in vitro digestibility analysis indicates that the plausible glycemic response of cassava flour revealed a stronger dependency on SEI than EFS under the influence of PEF. However, no difference was observed for the SDS fraction for all treatments (Table 2). A similar increase in RDS and decrease in both SDS and RS was also reported for waxy rice starch suspension at 40–50 kV/cm [3], while RS did not alter significantly for pea and japonica rice starch when treated at 2.86–8.57 kV/cm in solid-state [41].

Overall, the present study showed that PEF treatment enhanced the susceptibility of inherent starch in cassava flour to digestive enzymes, producing more glucose than native starch when digested but without affecting the rate of starch digestion (*k*) at the small intestinal phase and SDS fraction. The effect of PEF at mild intensity combined with elevated levels of specific energy input on the in vitro digestibility of the starch will help explore suitable application for PEF-treated cassava flour and the probability of creating novel products with tailored digestibility. At present, this is the first report on starch digestibility of PEF-treated cassava flour.

### 3.3. Effect of PEF on the Morphology and Birefringence of Cassava Starches in Cassava Flours

**Figure 2:**
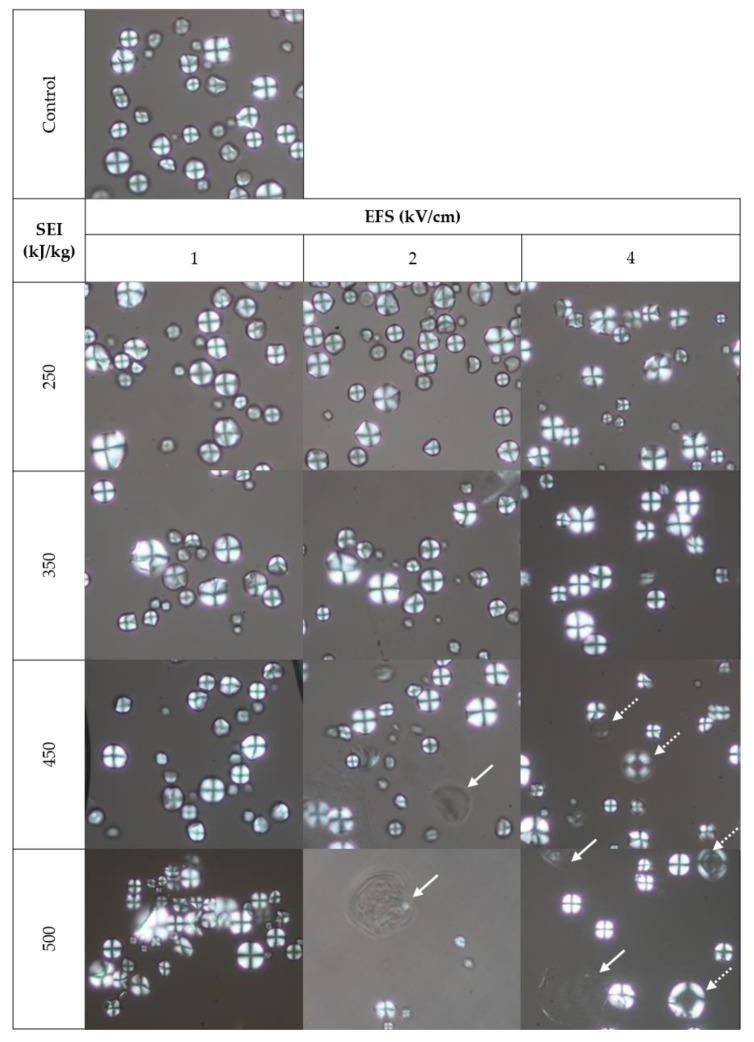
Polarized micrographs at 400× of “control” and PEF-treated cassava flour starches. Images show swollen starch granules with complete (solid arrow) and partial loss of birefringence (dashed arrow).

Starches from the flour samples showed typical cassava starch morphology (oval in shape with a truncated side) and a wide range of sizes [42]. There were few distorted and damaged granules observed even in the control that was potentially sustained during the flour milling process. Control flour samples were not visibly different to all PEF 1 combinations, along with PEF 2 and PEF 4 at 250–350 kJ/kg SEI. However, swollen starches were observed at PEF 2 and PEF 4 at 450–500 kJ/kg SEI (solid arrow; Figure 2). These were also present at 2–4 kV/cm combined with 418–484 kJ/kg energy input in oat flour [17] and gel-like structures in starches when treated at a higher intensity (40–50 kV/cm) [4,12]. The enlargement of starch indicates water uptake that occurs with gelatinization. Biliaderis [43] reported that if starch is heated in excess water progressively to higher temperatures, it takes up water and begins to swell irreversibly. However, PEF-treated flours after treatment have never reached onset gelatinization temperatures known for cassava starches (Table 1). However, mathematical models predict that higher temperatures may arise near the chamber wall undetected [44]. Conversely, it should also be taken into consideration that PEF applies short pulses (μs) of high-voltage and depending on the frequency used, any temperature increase in the sample will be intermittent and short during pulse delivery [17]. Moreover, the temperature increase will dissipate and equilibrate immediately with the surrounding temperature during the time gap between pulses.

**Figure 3:**
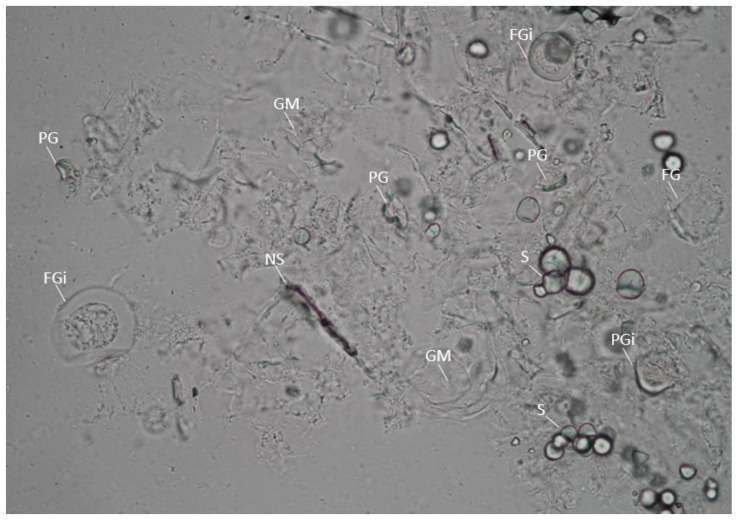
Micrograph at 400× of gelatinous layer produced after PEF 2 at 500 kJ/kg treatment of cassava flour. Photo reveals a gelatinous matrix (GM) with visibly entrapped intact (FGi) and disintegrated fully gelatinized granule (FG), intact (PGi) and disintegrated partially gelatinized granule (PG), native starch (S) and non-starch (NS) objects.

A few of the highly swollen starches showed granular disintegration. These aggregated with presumably hydrated non-starch components, thereby trapping other non-gelatinized or partially gelatinized starches (Figure 3). The congregation was evident in PEF 2 and 4 at 500 kJ/kg, as a floating gelatinous layer was observed after PEF treatment. It is postulated that granules lose the protection of the envelope, thus swelling easily. Consequently, van der Waals forces and electrostatic force between the granules would be strong enough to penetrate the boundaries of each other, causing aggregation [12]. Observations were consistent with the visible loss of granular shape, the appearance of surface pits, dissociation, and congregation of the fragments in previous works with starch from other plant sources; although a higher EFS (30–50 kV/cm) was used with no SEI reported [3,12]. Laser scattering techniques also detected an increase in particle size in the starches, as well as in oat flour treated at 2–4 kV/cm combined with 418–484 kJ/kg [11,12,17]. Cassava starch (tapioca), showed similar morphological changes when treated at 40 and 50 kV/cm with a calculated energy input of 51.29 and 80.14 kJ/kg, respectively [4]. On the other hand, Maniglia et al. [45] treated cassava starch at 15–25 kV/cm with a total specific energy input ranging from 25–50 kJ/kg and found no damage on the granule’s surface. In this study, inherent starch from the PEF-treated cassava flour did not show visible changes at 1 kV/cm up to 500 kJ/kg and until 350 kJ/kg at ~2–4 kV/cm. This clearly suggests a critical role of EFS and SEI, with higher intensity (40–50 kV/cm) requiring a lower SEI than mild intensities (2–4 kV/cm) to change the morphological characteristics of starch.

Polarized images revealed a Maltese cross pattern on the granule, as starch is birefringent owing to the natural assembly of amylose and amylopectin in the form of semi-crystalline granules [42]. Figure 2 reveals warping of the cross pattern for some granules with morphological distortion, but the cross intersection remains sharp and narrow. In contrast, the highly enlarged granules, resembling that of a “ghost” starch granule, had lost their Maltese cross (solid arrow; Figure 2), while others show birefringence in the periphery of the granule with a dark hallow center (dashed arrow; Figure 2). These were observed at PEF 2 and 4 at 450 kJ/kg and slightly more at 500 kJ/kg but not in all PEF 1 combinations and PEF 2 and 4 at ≤350 kJ/kg. The birefringence is related to the crystallinity of starch and its loss to disorganization [43]. Evidently, the PEF 2 and 4 at 450–500 kJ/kg are capable of changing the crystalline structure of starch to a more amorphous one, but not at a lower intensity. Conversely, when cassava starch was subjected to higher intensities of 15–25 kV/cm with a specific energy input ranging from 25–50 kJ/kg, a slight reduction in luminance of the cross was exhibited [45]. In brief, this study was able to show EFS and SEI dependency on morphology damage and loss of birefringence that seemed progressive with both elevated intensity and energy input.

### 3.4. Effect of PEF on Short-Range Microstructural Order of Cassava Starches in Cassava Flours

PEF 2 at 500 kJ/kg and 4 at 450–500 kJ/kg had a significantly lower 1047/1022 value than the control sample, while other treatments were not different from the control (Table 3). The higher the 1047/1022 absorbance ratio value, the more crystalline the starch is [34]. This indicates that these combinations shifted the microstructure into a more amorphous state, especially at higher EFS and SEI. PEF was able to transform starch into a non-crystal configuration by offering higher energy for the reaction between starch molecule chains and water molecules through hydrogen bond formation [3]. The significant reduction in the 1047/1022 absorbance ratio value was also reported for raw oat flour at 4.1 kV/cm and 441 kJ/kg [17], but for pea starch which has a similar diffractive pattern as cassava (C polymorph), an increase was observed at an EFS of 2.86 and 5.71 kV/cm [13]. It may be that other processing conditions and the nature of sample preparation can contribute to the difference in the degree of PEF response, i.e., cassava flour and raw oat flour were processed at higher hydration levels and a wider pulse width than the pea starch. It should also be noted that specific energy input was not reported for other works on starches; hence, a direct comparison cannot be made. Nonetheless, there was a clear indication of microstructure re-arrangement leading to a reduction in the 1047/1022 absorbance ratio value at 2 and 4 kV/cm with SEI greater than 450 kJ/kg.

Whereas no significant difference was found for 1022/995 absorbance ratio. Notably, PEF 2 and 4 at 500 kJ/kg had the highest average value. For 1022/995, a higher number means lower molecular order of starch double helices [36]. This may suggest that hydration potentially started in the amorphous layers of the starch granule, hence, minimal disruption of the double helices in the crystalline layers was detected. It should be considered that FTIR-ATR was reported to penetrate about ~2 μm from the surface [46]. As cassava starch diameter ranges from 2–35 μm [42,47,48], with an average size ranging between ~9.5–13.6 μm [49], the short-range order was most likely surficial. Moreover, Błaszczak et al. [50] found in potato starch a dense outer layer making it resistant to any changes. Thus, it can be inferred that the result found in the molecular order of the double helices was mostly from the external part of starch [46].

### 3.5. Effect of PEF on Gelatinization Temperatures and Enthalpy of Cassava Flours

In general, there was a shift to higher gelatinization temperatures and reduction in gelatinization enthalpy with the application of PEF at 2–4 kV/cm and at an elevated SEI (Table 4). PEF 4 at 500 kJ/kg was significantly higher for the onset and conclusion of gelatinization temperatures than the control and all PEF 1 combinations. Although, it was not different to PEF 2 at 500 kJ/kg and PEF 4 at 450 kJ/kg for Tonset, and to PEF 2 at ≥ 450 kJ/kg and PEF 4 at ≥350 kJ/kg for Tconclusion. Similarly, both raw and thermally processed oat flour also exhibited an increase in transition temperatures after PEF treatment, specifically at ~2 and ~4 kV/cm with 441–484 kJ/kg and 434 kJ/kg, respectively [17]. The result also showed a progressive effect on gelatinization transition temperatures, wherein it required less specific energy input to shift the temperature when the electric field strength was increased. However, the effect of increasing SEI at constant EFS on gelatinization temperatures has never been reported for pure starch. To date, only the work by Duque et al. [17] on oat flour considered the combined effects of SEI and EFS. This aside, the shift to higher gelatinization temperatures indicates the loss of the less stable crystalline structures [51] during PEF, leaving more uniform and perfect crystallites. Other authors have also suggested that the granular aggregation and physical reorganization inside the granule provided stability during heating [52], thereby requiring higher temperatures to facilitate starch swelling [17,21]. Furthermore, this shift did not impose much difference between control and treated samples in terms of Tpeak. In contrast, pure cassava starch showed a progressive decrease in gelatinization temperatures with increasing EFS [4,45]. This was also observed in starches from other sources and crystalline patterns [3,11,12,28]. Since only limited work has reported the effect of PEF on flour’s transition temperatures, the potential influence of non-starch components in the difference between flour and starch’s performance could be considered in the future. For Trange, no significant difference was found between the control and most of the treated samples. However, cassava samples treated with PEF 2 at 450 kJ/kg exhibited a significant lower Trange than cassava samples subjected to PEF 4 at 500 kJ/kg. According to Hublin [53] as cited by Han et al. [28], Trange reflects the degree of cohesion between crystallites, with stronger cohesion when Trange decreases. The narrowing of Trange as EFS was increased in maize starch, suggested the presence of crystallites of homogeneous stability, which allowed the fusion of crystallites of low cohesion and strengthened of the interactions between the remaining crystallite chains [28]. This agrees with the observed shift to higher gelatinization temperatures for PEF-treated cassava flours, which was attributed earlier to the loss of the less stable crystalline structures, thus producing more homogenous crystallites. However, increasing the SEI to 500 kJ/kg broadened the Trange of the PEF-treated cassava flour, especially at PEF 4. This result is likely suggests a weaker cohesion in the crystallites due to the disruption of molecular order that occurred along with PEF-assisted gelatinization. To note, PEF 4 at 500 kJ/kg led to cassava flour with the highest degree of gelatinization and the least ∆H. Other thermal properties also showed that PEF 4 at 500 kJ/kg had the highest Tpeak to Tonset difference, which led to a higher Trange.

Nonetheless, a decrease in the gelatinization enthalpy (ΔH) with increasing EFS as observed in this study agrees with previous work on PEF-treated pure starches [3,11,12,28,41]. The effect of increasing energy input that comes with increasing EFS on cassava starch also showed a reducing trend on ΔH [4]. This suggests a lower energy requirement to melt the crystalline amylopectin structure and the double helices of amylose of PEF-treated starch samples [45], during domestic or industrial thermally induced gelatinization. The magnitude of enthalpy change is also associated with disruption of ordered structure that occurs with gelatinization [54]. This study was able to show that at constant EFS with sufficient SEI, gelatinization can progress. Additionally, a strong correlation was found between ΔH and EFS in japonica rice starch [41], as well as a positive relationship between ΔH and relative crystallinity in oat flour fractions [21].

The effect on ΔH corroborates with the degree of gelatinization result, wherein PEF 4 at 500 kJ/kg > PEF 2 at 500 kJ/kg > PEF 2 at 450 kJ/kg and PEF 4 at 450 kJ/kg > other treatment combinations. In summary, the results showed that the microstructural integrity of cassava starch was reduced from SEI 450 kJ/kg and upwards, with increasing impact at higher EFS. This was supported by the polarized images showing loss of birefringence, reduction in the 1047/1022 absorbance ratio value, and decreasing ΔH. However, at a lower EFS of 1 kV/cm, increasing the SEI up to 500 kJ/kg was unable to induce changes in the flour or in its inherent starch. Further study is necessary to study the critical SEI that could promote a shift in thermal properties at this intensity.

## 4. Conclusions

In conclusion, the findings from this study demonstrated that PEF treatment enhanced the starch digestibility of cassava flour. In particular, an increase in the RDS, decrease in RS, and no changes in the SDS fraction and rate of starch digestion in the small intestinal phase can be achieved with mild electric field strength but at high-energy input for cassava flour. Detailed investigation has shown that PEF treatment caused morphological damage and transformed the short-range structure of inherent cassava starch to a more amorphous one through gelatinization; thereby shifting the gelatinization temperatures to higher temperatures and reducing the enthalpy change. At mild intensities (2–4 kV/cm), PEF was able to induce changes in the flour’s starch properties, which were formerly achieved at higher electric field strengths for pure starches (25–50 kV/cm) but given sufficient specific energy input is applied (≥450 kJ/kg). This study further establishes that PEF can be utilized as a nonthermal and clean technology to change cassava flour’s starch digestibility and its related properties. However, potential new applications delivered by PEF require a detailed cost–benefit analysis to be conducted before recommending its adoption at commercial scale. The difference in magnitude of PEF impact on flour from previous reports further supports the claim that electric field strength and specific energy input are important processing factors to report in order to achieve reproducible results, along with inherent sample factors and other processing conditions. Further research on comparing flour and its isolated starch would be helpful to decrease this information gap.

## Figures and Tables

**Table 1:** Summary of processing parameters and temperature of cassava flour suspension observed during PEF treatment.

Sample	EFS (kV/cm)	SEI (kJ/kg)	Pulse Number	Initial Temperature (°C)	Final Temperature (°C)
PEF 1	1.02	249.7 ± 0.1	14,584	13.3 ± 0.6	34.3 ± 1.3
	1.05	349.3 ± 0.4	20,417	11.1 ± 0.7	37.1 ± 0.7
	1.05	452.0 ± 4.1	26,250	9.9 ± 0.5	39.3 ± 0.9
	1.05	499.2 ± 0.2	29,167	8.2 ± 0.1	38.2 ± 0.4
PEF 2	2.00	250.4 ± 2.0	3210	10.8 ± 0.3	34.0 ± 0.1
	2.00	348.8 ± 1.7	3990	11.7 ± 0.9	40.5 ± 1.1
	1.95	449.7 ± 0.1	4777	12.4 ± 0.1	46.3 ± 1.3
	1.95	498.6 ± 1.4	5148	12.7 ± 0.3	48.7 ± 0.2
PEF 4	4.03	249.9 ± 0.7	724	11.9 ± 0.6	34.1 ± 1.1
	3.99	350.7 ± 0.6	920	12.1 ± 0.2	40.8 ± 0.8
	3.96	449.1 ± 2.0	1117	11.1 ± 0.4	46.1 ± 0.9
	3.95	504.2 ± 4.3	1203	10.3 ± 0.4	48.4 ± 0.3

Results are presented as mean (±standard deviation) of individual PEF treatment (n = 30).

**Table 2:** Starch digestion fractions and average rate of starch digestion in the small intestinal phase of cassava flour after PEF treatment.

Sample	Electric Field Strength (kV/cm)	Specific Energy Input (kJ/kg)	RDS (%)	SDS (%)	RS (%)	*k* (×10^−2^ min^−1^)	Range of R^2^ for *k* estimation
Control	-	-	1.89 ± 0.06 ^c^	1.45 ± 0.16 ^ab^	96.66 ± 0.22 ^ab^	10.76 ± 1.00 ^ab^	0.95–0.99
PEF 1	1.02	249.7 ± 0.1	1.64 ± 0.14 ^c^	1.53 ± 0.04 ^a^	96.83 ± 0.18 ^a^	11.74 ± 1.51 ^ab^	0.97–0.98
	1.05	349.3 ± 0.4	1.88 ± 0.02 ^bc^	1.51 ± 0.36 ^ab^	96.61 ± 0.34 ^ab^	12.52 ± 1.97 ^ab^	0.94–0.98
	1.05	452.0 ± 4.1	1.99 ± 0.14 ^bc^	1.36 ± 0.06 ^a^	96.64 ± 0.08 ^ab^	11.88 ± 0.19 ^ab^	0.97–0.99
	1.05	499.2 ± 0.2	2.19 ± 0.41 ^bc^	1.65 ± 0.09 ^a^	96.16 ± 0.32 ^ab^	12.53 ± 1.29 ^ab^	0.93–0.97
PEF 2	2.00	250.4 ± 2.0	1.97 ± 0.08 ^bc^	1.49 ± 0.35 ^ab^	96.54 ± 0.43 ^ab^	12.69 ± 0.41 ^ab^	0.97–0.98
	2.00	348.8 ± 1.7	2.35 ± 0.09 ^b^	1.73 ± 0.10 ^a^	95.92 ± 0.12 ^b^	13.07 ± 0.70 ^a^	0.94–0.97
	1.95	449.7 ± 0.1	5.06 ± 0.29 ^a^	1.29 ± 0.26 ^ab^	93.65 ± 0.51 ^cd^	12.27 ± 0.47 ^ab^	0.92–0.93
	1.95	498.6 ± 1.4	6.22 ± 0.35 ^a^	1.04 ± 0.07 ^b^	92.75 ± 0.28 ^e^	11.09 ± 0.15 ^ab^	0.86–0.90
PEF 4	4.03	249.9 ± 0.7	1.90 ± 0.09 ^c^	1.58 ± 0.07 ^a^	96.52 ± 0.11 ^ab^	11.86 ± 0.40 ^ab^	0.97–0.98
	3.99	350.7 ± 0.6	2.13 ± 0.34 ^bc^	1.44 ± 0.13 ^ab^	96.43 ± 0.40 ^ab^	11.70 ± 0.43 ^ab^	0.97–0.98
	3.96	449.1 ± 2.0	4.84 ± 0.39 ^a^	1.18 ± 0.22 ^ab^	93.98 ± 0.24 ^c^	10.81 ± 0.82 ^ab^	0.85–0.88
	3.95	504.2 ± 4.3	6.05 ± 0.17 ^a^	1.03 ± 0.32 ^ab^	92.93 ± 0.19 ^de^	9.88 ± 1.29 ^b^	0.69–0.90
*p*-value			*< 0.01*	*< 0.01*	*< 0.01*	*0.021*	

RDS: rapidly digestible starch; SDS: slowly digestible starch; RS: resistant starch; *k*; average of rate of starch digestion in the small intestinal phase. Means (± standard deviation; n = 3) with similar letters per column are not significantly different.

**Table 3:** Absorbance ratio values of control (untreated) and PEF-treated cassava flours.

Sample	Electric Field Strength (kV/cm)	Specific Energy Input (kJ/kg)	Abs 1047/1022 *	Abs 1022/995 ^ns^
Control	-	-	0.799 ± 0.055 ^a^	0.787 ± 0.083
PEF 1	1.02	249.7 ± 0.1	0.778 ± 0.082 ^a^	0.834 ± 0.047
	1.05	349.3 ± 0.4	0.787 ± 0.103 ^a^	0.897 ± 0.158
	1.05	452.0 ± 4.1	0.748 ± 0.047 ^ab^	0.833 ± 0.008
	1.05	499.2 ± 0.2	0.730 ± 0.030 ^abc^	0.857 ± 0.049
PEF 2	2.00	250.4 ± 2.0	0.759 ± 0.052 ^a^	0.820 ± 0.045
	2.00	348.8 ± 1.7	0.750 ± 0.113 ^ab^	0.820 ± 0.008
	1.95	449.7 ± 0.1	0.601 ± 0.039 ^abcd^	0.847 ± 0.065
	1.95	498.6 ± 1.4	0.511 ± 0.065 ^d^	0.951 ± 0.132
PEF 4	4.03	249.9 ± 0.7	0.748 ± 0.026 ^ab^	0.864 ± 0.081
	3.99	350.7 ± 0.6	0.782 ± 0.016 ^a^	0.837 ± 0.039
	3.96	449.1 ± 2.0	0.545 ± 0.043 ^cd^	0.802 ± 0.039
	3.95	504.2 ± 4.3	0.563 ± 0.082 ^bcd^	0.912 ± 0.057

* significant at 0.01% alpha level; ^ns^ not significant at 0.05% alpha level. Means (± standard deviation; n = 3) with similar letters per column are not significantly different.

**Table 4:** Gelatinization transition properties of PEF-treated cassava flours determined through DSC.

Sample	Electric Field Strength (kV/cm)	Specific Energy Input (kJ/kg)	Tonset	Tpeak	Tconclusion	Trange	∆H (J/g)	Degree of Gelatinization (%)
Control	-	-	63.07 ± 0.22 ^c^	68.04 ± 0.33 ^ab^	73.43 ± 0.30 ^b^	9.95 ± 0.63 ^ab^	3.75 ± 0.19 ^a^	-
PEF 1	1.02	249.7 ± 0.1	63.44 ± 0.21 ^c^	68.26 ± 0.11 ^b^	73.56 ± 0.17 ^b^	9.64 ± 0.39 ^abc^	3.77 ± 0.11 ^a^	−0.47 ± 2.84 ^d^
	1.05	349.3 ± 0.4	63.56 ± 0.36 ^c^	68.45 ± 0.23 ^ab^	74.06 ± 0.21 ^b^	9.79 ± 0.27 ^abc^	3.87 ± 0.16 ^a^	−3.02 ± 4.19 ^d^
	1.05	452.0 ± 4.1	63.84 ± 0.45 ^bc^	68.67 ± 0.60 ^ab^	73.85 ± 0.31 ^b^	9.66 ± 0.34 ^abc^	3.73 ± 0.19 ^a^	0.65 ± 4.89 ^d^
	1.05	499.2 ± 0.2	63.48 ± 0.55 ^c^	68.26 ± 0.56 ^ab^	73.78 ± 0.69 ^b^	9.56 ± 0.06 ^abc^	4.01 ± 0.30 ^a^	−6.68 ± 7.86 ^d^
PEF 2	2.00	250.4 ± 2.0	63.79 ± 0.32 ^bc^	68.47 ± 0.11 ^b^	73.94 ± 0.21 ^b^	9.36 ± 0.46 ^bc^	3.70 ± 0.12 ^a^	1.55 ± 3.15 ^d^
	2.00	348.8 ± 1.7	63.14 ± 0.40 ^c^	67.83 ± 0.46 ^ab^	73.41 ± 0.48 ^b^	9.38 ± 0.24 ^bc^	3.91 ± 0.18 ^a^	−4.07 ± 4.58 ^d^
	1.95	449.7 ± 0.1	63.91 ± 0.28 ^bc^	68.21 ± 0.12 ^b^	74.44 ± 0.26 ^ab^	8.62 ± 0.49 ^c^	2.94 ± 0.11 ^b^	21.63 ± 2.82 ^c^
	1.95	498.6 ± 1.4	64.90 ± 0.48 ^ab^	69.84 ± 0.77 ^ab^	76.09 ± 0.74 ^ab^	9.89 ± 0.62 ^abc^	2.23 ± 0.03 ^c^	40.67 ± 0.57 ^b^
PEF 4	4.03	249.9 ± 0.7	63.96 ± 0.55 ^bc^	68.76 ± 0.58 ^ab^	74.24 ± 0.36 ^b^	9.60 ± 0.19 ^abc^	4.03 ± 0.17 ^a^	−7.44 ± 4.34 ^d^
	3.99	350.7 ± 0.6	63.80 ± 0.28 ^bc^	68.44 ± 0.12 ^b^	74.01 ± 0.15 ^ab^	9.30 ± 0.37 ^bc^	3.87 ± 0.06 ^a^	−3.14 ± 1.39 ^d^
	3.96	449.1 ± 2.0	64.75 ± 0.25 ^ab^	69.19 ± 0.16 ^a^	74.93 ± 0.76 ^ab^	8.88 ± 0.43 ^bc^	2.89 ± 0.15 ^b^	22.95 ± 3.83 ^c^
	3.95	504.2 ± 4.3	65.56 ± 0.42 ^a^	70.91 ± 0.70 ^ab^	77.91 ± 0.69 a	10.72 ± 0.78 ^a^	1.40 ± 0.22 ^d^	62.88 ± 5.64 ^a^
*p*-value			*<0.001*	*0.002*	*<0.001*	*0.001*	*<0.001*	*<0.001*

T: temperature (°C); ΔH: gelatinization enthalpy. Means (± standard deviation; n = 3) with similar letters per column are not significantly different.

## Data Availability

Data is contained within the article.
